# Vacuum level for opening the streak canal and measurement of machine-induced changes in teat tissue during milking of dairy camels (*Camelus dromedarius*)

**DOI:** 10.3389/fvets.2025.1576902

**Published:** 2025-04-01

**Authors:** Moufida Atigui, Marwa Brahmi, Madeh Sadan, Fahad A. Alshanbari, Khouloud Dahmani, Wiem Ben Salem, Mohamed Hammadi, Pierre-Guy Marnet

**Affiliations:** ^1^Livestock and Wildlife Laboratory, Arid Regions Institute, IRESA, Medenine, Tunisia; ^2^Institution de la Recherche et de l’Enseignement Supérieur Agricoles, Tunis, Tunisia; ^3^Higher Institute of Agricultural Science of Chott Mariem, Sousse, Tunisia; ^4^Department of Clinical Sciences, College of Veterinary Medicine, Qassim University, Buraidah, Saudi Arabia; ^5^Department of Medical Biosciences, College of Veterinary Medicine, Qassim University, Buraidah, Saudi Arabia; ^6^Office de l’Elevage et des Pâturages, Tunis, Tunisia; ^7^Ecole Doctorale SIS, Université de Gabès, Gabès, Tunisia; ^8^Department of Animal and Food Sciences, Institut Agro Rennes-Angers, Rennes, France; ^9^UMR SELMET, CIRAD, INRAe, Institut Agro, Montpellier, France

**Keywords:** machine milking, vacuum level, teat sphincter, cutimeter, diagnostic imaging, camels

## Abstract

This research aims to study some of the teat characteristics involved in milkability of dairy camels, including the relationship between teat anatomy and the vacuum needed to open the teat sphincter (VLOTS). It also investigates short-term machine milking-induced changes in teat tissue thickness and teat anatomical characteristics, as well as their implications for udder health in dairy camels. To study VLOTS, 10 dairy camels in mid-lactation (weight: 516.6 ± 19 kg; age: 13.4 ± 3.8 years; parity: 5 ± 1.8; average milk yield: 7.3 ± 0.8 L/day) were used in Experiment 1. VLOTS was measured 4 h after morning milking for all four teats using an apparatus called a vacuumeter, without a liner or pulsation. Measurements were repeated three times at 2-day intervals and considered as repetition. Teat canal length (TCL), teat wall thickness (TWT), teat cistern separation wall thickness (TSWT), teat cistern diameter (TCD), and teat length were measured using ultrasound. Experiment 2 was performed on six dairy camels in late lactation (weight: 460 ± 55.15 kg; age: 10.2 ± 2.4 years; parity: 3.5 ± 1.0; average milk yield: 3.6 ± 0.7 L/day). External teat measurements (length, barrel, and apex diameters) were recorded with a caliper. Pre-and post-milking teat-end thickness (TET) were evaluated with a cutimeter at 2 cm from the teat end. Ultrasound imaging was performed pre-and post-milking to determine TCL, TWT, TCD, and teat apex diameter (TAD). Milk ejection time and total milking duration were recorded. Residual milk volume was estimated after an injection of 10 IU oxytocin. Milk samples were taken for somatic cell count (SCC). The results showed that only 50 teats out of 120 observations exhibited milk flow at a vacuum up to 70 kPa. Teats were divided into three groups: Group 1 included easy-opening teats that opened at a vacuum level of less than 30 kPa; group 2 included teats that were hard to open, requiring a higher vacuum (31 up to 69 kPa); and group 3 included teats that did not show milk flow at a vacuum higher than 70 kPa. The mean VLOTS for groups 1 and 2 were 19.39 ± 0.66 kPa and 47.13 ± 2.14 kPa, respectively. VLOTS was positively and highly correlated with TCL, TWT, and TSWT (*r* = 0.71, *r* = 0.62, and *r* = 0.51, respectively, *p* < 0.001) and negatively correlated with teat length and diameter (*r* = −0.50 and *r* = −0.30, respectively, *p* < 0.01). Observation of teats immediately after cluster removal revealed a 15.4% decrease in TET. TCL and TWT increased by 20.3 and 40.5%, respectively, while TCD and TAD decreased by 40.3 and 19.9%, respectively, after milking. This suggests the stretching of the teat extremity and congestion of the teat barrel wall. The mean SCC recorded in this study was 149.6 10^3^ cells/mL, varying from 37.5 10^3^ cells/mL to 287.5 10^3^ cells/mL. This study confirms the need for a high vacuum level to overcome the sphincter barrier in dromedary camels. However, it suggests the deleterious effect of large camel teats in a cow’s liner.

## 1 Introduction

Efficient and sustainable milk withdrawal from camels’ (*Camelus dromedarius*) udders remains a cornerstone for optimizing machine milking and enhancing the economic viability of dairy camel farming. Understanding the animal/machine interaction is critical for adapting machine milking to the various situations encountered worldwide and to the specific needs of different animals (1). Unlike bovine species, the anatomy of the camel’s mammary gland and the dynamics of milking remain underexplored (2–4). The streak canal acts as the primary barrier against microbial invasion while facilitating milk emission during milking. Its functionality is influenced by various factors, including vacuum levels applied during milking. Insufficient or excessive vacuum pressure can either increase the probability of liner slips because the adhesion of the liner to the teat is reduced or cause tissue damage, compromising udder health (5, 6). Therefore, determining the optimal vacuum level for opening the streak canal is important for developing milking protocols that balance milk yield, efficiency, and animal welfare (7, 8). Furthermore, the response of teat-end thickness to used parameters or to the liner of milking equipment is also a good tool for the study of milkability and the measurement of machine-induced changes in teat tissue during milking in dairy animals. The impact of machine milking on the teat tissue is inevitable and can be sorted into short-, medium- and long-term effects (9–11). Short-term reversible teat changes including color changes, firmness, thickness, and openness of the teat orifice are commune (12). However, only repeated or prolonged exposure to improper milking conditions causing excessive load on the teat can lead to long-term alterations in teat tissue structure that become hyperkeratosis and potentially increasing susceptibility to mastitis (13–15). Differences in short-term implications for milking efficiency or udder health have been attributed to the variation in anatomical dimensions and structures among teats with different teat-end shapes (9, 16). Thus, understanding the teat’s functional anatomy, particularly the streak canal and its interaction with the milking machine, is crucial for improving milking efficiency while maintaining udder health and animal welfare (15). In dairy species, considerable research has been conducted on the effects of the milking liner on the teat tissue integrity (9, 10, 17, 18). However, in dairy camels, such insights remain scarce, necessitating targeted studies to fill this knowledge gap. This research aims to investigate the vacuum levels required to open the streak canal effectively in dairy camels and explore the machine-induced short-term changes in teat tissue during milking.

## 2 Materials and methods

### 2.1 Animals, housing, and management

The trial was carried out in the experimental farm of the Arid Regions Institute (IRA, Chenchou, Tunisia). Clinically healthy Maghrebi dromedary camels free from clinical mastitis and have an udder without abnormalities such as non-lactating quarters or teat injuries, and edema were used for the experiments. Animals were housed in a free-stall barn. For the first experiment, camels received/head 5 kg of fresh lucerne (dry matter, DM 17.2%; crude protein, CP, 17.9%; neutral detergent fiber, NDF, 39.6.0%; net energy for lactation, NEL, 1.53 Mcal/kg; on a DM basis), 7 kg of oat hay (DM, 84.6%; CP, 8.4%; NDF, 68.2%; NEL, 0.71 Mcal/kg; on DM basis), 3 kg of lucerne pellets (DM,90.4%; CP, 19.6%; NDF, 64.2%; NEL, 1.25 Mcal/kg on DM basis), and 2 kg of commercial concentrate (DM 91.0%; CP 17.0%; NDF 22.2%; NEL 1.53 Mcal/kg; on a DM basis).

Camels in the second experiment were fed (/head) with a forage mixture of 5 kg of lucerne hay (DM, 96.6%; CP, 17.1%; NDF, 47.3%; NEL, 1.42 Mcal/kg; on a DM basis) and 7 kg of oat hay (DM 96.0%; CP 9.7%; NDF 70.3%; NEL 0.58 Mcal/kg; on a DM basis), supplemented with 2 kg of a commercial concentrate (DM 91.0%; CP 17.0%; NDF 22.2%; NEL 1.53 Mcal/kg; on a DM basis). Diets were calculated based on milk production and lactation stage of camels and available feed in the farm at the time of the experiment. All animals had free access to water.

### 2.2 Milking

Camels were milked in a 2 × 3 herringbone milking parlor (FLACO, Spain) once a day at the time of the experiment. The milking unit was equipped with a FLACO milking cluster (weight: 2.1 kg; claw volume: 240 cc). The milking liner was rubber liner type ULTRAmilk, JD317 (mouthpiece bore diameter, 22.5 mm; mouthpiece depth, 27 mm; barrel shape, concave; barrel length, 155 mm). The machine was set at a 60 ppm pulsation rate with a 60:40 pulsation ratio and 48-kPa vacuum level. The pre-milking (cleaning, fore-stripping, and manual teat stimulation) lasted for 30 s; then clusters were attached. Cluster removal was manual when milk flow ceased and visually confirmed.

#### 2.2.1 Experiment 1: vacuum level to open teat sphincter

Ten dairy camels at mid-lactation (weight: 516.6 ± 19 kg; Age: 13.4 ± 3.8 years, parity: 5 ± 1.8; average daily milk yield was 7.3 ± 0.8 L/day) were used for the first experiment. The vacuum needed to open the teat sphincter (VLOTS) was determined with a prototype apparatus called a “vacuumeter,” a transparent teat cup without liner nor pulsation (19). The vacuum was then gradually increased from zero until milk flow started or to a maximum of 70 kPa. The teat sphincter was considered open when the first drop of milk emerged from the teat. VLOTS was recorded 4 h after morning milking for all four teats to prevent spontaneous milk ejection and limit the effect of high intra-mammary pressure. Measurements were repeated three times at 2-day intervals and considered as repetition. Teats’ characteristics were evaluated by ultrasound. After VLOTS evaluation, teats were divided into three groups where group 1 included easy-opening teats that opened at a vacuum level of less than 30 kPa, group 2 included teats hard to open that needed vacuum ranging between 31 and 69 kPa, and group 3 included teats that did not show milk flow at vacuum higher than 70 kPa.

#### 2.2.2 Experiment 2: milking-induced changes in the teat

Six multiparous Maghrebi camels (weight: 460 ± 55.15 kg; age: 10.2 ± 2.4 years; parity: 3.5 ± 1.0) well-trained to machine milking were selected for this trial. Camels were at late lactation with an average daily milk yield of 3.6 ± 0.7 L. The average teat length was 4.1 ± 0.1 cm for front teats and 4.6 ± 0.1 cm for rear teats. The teat barrel diameter for the front and rear teats was on average 3.3 ± 0.1 cm and 3.6 ± 0.1 cm, respectively. Teats’ shapes were cylindrical (68%), conical (17%), and sometimes irregular (15%). Teat external measurements (length, barrel, and apex diameters) were recorded with a caliper. Internal teat measurements were taken before and immediately after milking. Teat-end tissue thickness (TET) was measured before and after milking with a cutimeter at 2 cm of teat end. The cutimeter measurements were made with a spring-loaded caliper as described by Hamann et al. (20) and Marnet et al. (21). These measurements were performed after the ultrasound scans, so it would not affect the images. In this experiment, the jaws of the cutimeter were placed approximately 2 cm above the teat tip. Internal teat measurements were measured by ultrasonography as presented in the following section. Milking-induced changes were calculated as follows:


$$Milkinginducedchanges\%=\frac{\left(\begin{array}{l} \mathrm{post milking value}-\\ \mathrm{premilking value} \end{array}\right)\;}{\mathrm{premilking value}}\times 100$$

All measurements were taken immediately before and after morning milking by a trained researcher.

Milk ejection time and milking duration were recorded. Residual milk volume was estimated after an injection of 10 IU oxytocin, and samples of machine and residual milk fractions were taken for somatic cell count (SCC).

### 2.3 Ultrasound scanning

**Figure 1:**
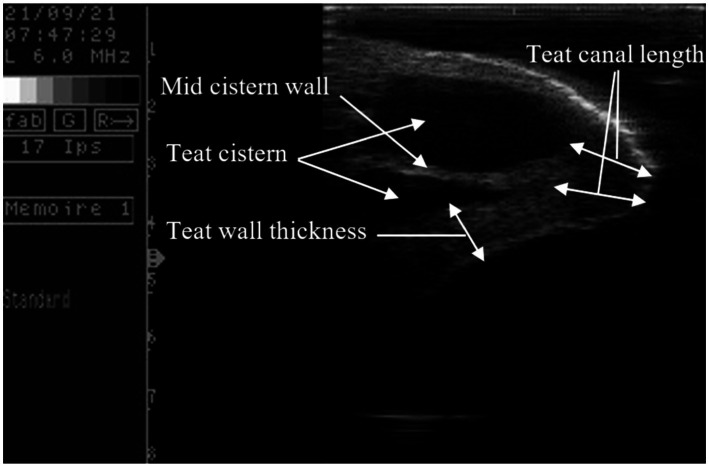
Measurements taken on camel teat ultrasound scan.

An ultrasound unit (Aquila Pro, e-saote piemedical, The Netherlands) equipped with a 6-MHz linear probe was used to measure the teat internal measurements and changes in the teat’s tissue. The ultrasound was performed in the milking parlor on both the front and rear teats on the operator’s side. The water bath method was used as described by Gleeson et al. (22) to prevent deformation and ensure the complete presentation of the teat. In brief, the teat was immersed in a plastic container containing warm water. The probe was coated with a film of lubricant gel and applied to the external surface of the container. A vertical cross-section of each teat was scanned in triplicates before VLOTS recording (*Experiment 1*), during pre-milking preparation, and immediately after cluster removal (*Experiment 2*). Longitudinal cross-sectional images were used for measurements (Figure 1) by means of image treatment software (Image Tool 3.00).

### 2.4 Somatic cell count

Milk SCC was counted using the direct microscopic method. In brief, an amount of 10 μL milk sample was spread over the Malassez slide. The smear was stained with methylene blue for 10 min, followed by somatic cell count via direct microscopic examination.

### 2.5 Statistical analysis

All analyses were performed using Mixed Procedures of SAS version 9.3 (SAS Institute Inc., Cary, NC). For the first trial, the models included the general mean, the random effect of the animal (1–10), the fixed effect of the teat position (front and rear), and the fixed effect of the VLOTS class (1–3) and the random error. For the second, the model included the general mean, the random effect of the animal (1–6), the fixed effect of the treatments (before and after milking), teat position (front and rear), and the random error. Differences between means were tested by the PDIFF test. The level of statistical significance was set at a *p*-value of <0.05, unless otherwise stated. All data are presented as means ± SEM.

The SCC showed a non-normal distribution and was transformed using a logarithm function. We used the formula proposed by Ali and Shook (23) as follows:


logSCC=log2(SCC/100000)+3

logSCC was then analyzed using the PROC mixed model. Pearson’s correlation was calculated to show possible relationships between teats’ characteristics and VLOST in the first experiment and teat characteristics, residual milk, milking times, and SCC in the second experiment.

## 3 Results

### 3.1 Vacuum level to open teat sphincter

The vacuum level of the milking machine required to open the teat sphincter (VLOTS) in camels varied widely both inter- and intra-animal. The continuously increasing vacuum (no liner, no pulsation) caused milk flow in only 50 out of 120 teats up to a vacuum of 70 kPa during this experiment. Teats were classed into three groups according to VLOTS level, where group 1 referred to easy-opening teats needing an average of 19.39 ± 0.66 kPa, group 2 required a mean of 47.13 ± 2.14 to observe the first milk drop, while in group 3, the teat sphincter did not open even when vacuum exceeded 70 kPa. Internal teat measurements according to VLOTS group are presented in Table 1. TCL, TWT, and TSWT were significantly shorter, while TCD and TL were significantly higher in easy-opening teats. Interestingly, camels with harder opening teats had significantly higher milk yield (*p* < 0.01). The effect of parity and teat position was tested, and no significant effect was detected.

**Table 1:** Overall means of vacuum level to open teat sphincter, evaluated internal teat measures, and milk yield according to levels of teat sphincter opening.

	Group 1	Group 2	Group 3
VLOTS, kPa	19.39 ± 0.66	47.13 ± 2.14	≥ 70
TCL, cm	0.71 ± 0.06^b^	1.43 ± 0.05^a^	1.46 ± 0.03^a^
TWT, cm	0.64 ± 0.04^b^	0.98 ± 0.05^a^	1.03 ± 0.03^a^
TAD, cm	2.16 ± 0.06	2.22 ± 0.07	2.09 ± 0.04
TCD, cm	4.03 ± 0.14^a^	3.89 ± 0.17^ab^	3.65 ± 0.07^b^
TL, cm	6.16 ± 0.14^a^	5.59 ± 0.21^b^	5.33 ± 0.10^b^
TSWT, cm	0.42 ± 0.02^b^	0.66 ± 0.04^a^	0.63 ± 0.04^a^
MMY, kg	4.01 ± 0.03^c^	5.07 ± 0.08^b^	5.77 ± 0.12^a^
DMY, kg	6.74 ± 0.09^c^	7.50 ± 0.08^b^	8.61 ± 0.23^a^

TCL, teat canal length; TWT, teat wall thickness; TAD, teat apex diameter; TCD, teat cistern diameter; TL, teat length; TSWT, teat cistern separation wall thickness; MMY, morning milk yield; DMY, daily milk yield; VLOTS, vacuum level to open teat sphincter. ^a,b,c^Means within a line with different superscripts are significantly different (*p* < 0.05).

Pearson’s correlation coefficient (Table 2) among internal teat measurements, milk yields, and VLOTS was strong and positive, except for the TCD and TL which showed a strong negative correlation with VLOTS. Teat internal traits showed a strong and positive correlation between TCL, TWT, and TSWT and were negatively correlated with TL. Only TCL and TSWT were correlated with milk yields, while TCD was correlated with only the daily milk yield.

**Table 2:** Correlation coefficient between studied internal teat’s traits, milk yields, and VLOTS.

	TCL	TWT	TAD	TSWT	TCD	TL	MMY	DMY
TCL	1							
TWT	0.69^***^							
TAD	0.01	0.16						
TSWT	0.56^***^	0.53^***^	0.08					
TCD	−0.13	0.03	0.72^***^	0.05				
TL	−0.29^*^	−0.20^*^	0.64^***^	−0.15	0.76^***^			
MMY	0.30^**^	0.18	0.14	0.26^*^	0.17	0.10		
DMY	0.28^*^	0.15	0.20	0.26^*^	0.27^*^	0.17	0.96^***^	
VLOTS	0.71^***^	0.62^***^	−0.19	0.51^***^	−0.30^**^	−0.50^***^	0.40^***^	0.33^**^

TCL, teat canal length; TWT, teat wall thickness; TAD, teat apex diameter; TCD, teat cistern diameter; TL, teat length; TSWT, teat cistern separation wall thickness; MMY, morning milk yield; DMY, daily milk yield; VLOTS, vacuum level to open teat sphincter. ^*^p < 0.05; ^**^p < 0.01; ^***^p < 0.0001.

### 3.2 Changes in the teat tissue parameters immediately after milking

**Figure 2:**
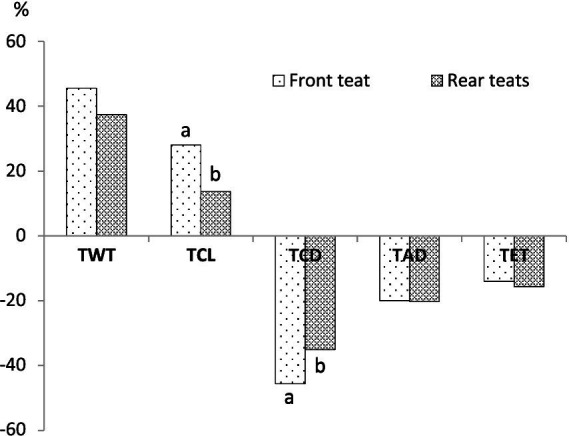
Average milking-induced change (%) in front and rear teats. TWT, teat wall thickness; TCL, teat canal length; TCD, teat cistern diameter; TAD, teat apex diameter; TET, teat-end thickness. ^a,b^Columns with different superscripts are significantly different (*p* < 0.05).

Average teat internal measurements before and immediately after milking are presented in Table 3, while the relative changes induced by milking according to the teat position are illustrated in Figure 2. TWT increased by 40.5 ± 4.3%, TCL increased by 20.3 ± 5.3%, while TCD and TAD decreased by 40.3 ± 8.2% and 19.9 ± 7.8%, respectively. Teat position affected only TCL and TCD with a significantly stronger effect (*p* < 0.001) observed in front teats. TET decreases by 15.4 ± 1.1% with no difference according to teat position.

**Table 3:** Effect of machine milking on average teat tissue measurements (cm) determined by ultrasonography and cutimeter before and immediately after milking.

	Before milking	After milking
Teat wall thickness	1.00 ± 0.26^b^	1.41 ± 0.36^a^
Teat canal length	1.29 ± 0.62^b^	1.56 ± 0.51^a^
Teat cistern diameter	2.71 ± 0.67^a^	1.62 ± 0.61^b^
Teat apex diameter	2.70 ± 0.31^a^	2.17 ± 0.24^b^
Teat-end thickness	1.3 ± 0.26^a^	1.1 ± 0.10^b^

^a,b^Means within a line with different superscripts are significantly different (*p* < 0.05).

Pearson’s coefficients calculated between internal and external teat measurements (Table 4) showed negative correlations between TET measured by cutimeter and TWT and TCL with *r* = −0.21, *p* < 0.05 and *r* = −0.36, *p* < 0.0001, respectively. Similarly, TCD was strongly and negatively correlated with TWT and TCL (*r* = −0.50; *p* < 0.0001 and *r* = −0.45; *p* < 0.0001, respectively). Furthermore, TET was positively correlated with all external teat measurements, whereas TCL was negatively correlated with external teat diameter measured at different levels (apex, barrel, and basal levels) and positively correlated with TL. Moreover, the mean SCC recorded in this study was 149.6 10^3^ cells/mL and was positively correlated with TCL (*r* = 0.21; *p* < 0.01) and negatively correlated with TAD (*r* = 0.20; *p* < 0.05). Residual milk, time to milk ejection, and total milking duration were not correlated with the teat characteristics, except for TL, which was positively correlated with milk ejection time and total milking duration (*r* = 0.35; *p* < 0.0001 and *r* = 0.22; *p* < 0.01, respectively) (Table 5).

**Table 4:** Pearson correlation coefficients between teat internal and external measurements.

	TWT	TCL	TCD	TET	TAD	TBsD	TBrD
TCL	0.29^**^						
TCD	−0.50^***^	−0.45^***^					
TET	−0.21^*^	−0.36^***^	0.31^**^				
TAD	−0.30^**^	−0.17	0.59^***^	0.41^***^			
TBsD	0.04	−0.55^***^	0.21^*^	0.29^**^	−0.01		
TBrD	0.28^**^	−0.46^***^	0.20^*^	0.27^**^	−0.01	0.61^***^	
TL	0.09	0.30^**^	−0.08	0.34^**^	0.20^*^	0.44^***^	0.22^**^

TWT, teat wall thickness; TCL, teat canal length; TCD, teat cistern diameter; TET, teat-end thickness; TAD, teat apex diameter; TBsD, teat basal diameter; TBrD, teat barrel diameter; TL, teat length. ^*^p < 0.05; ^**^p < 0.01; ^***^p < 0.0001.

**Table 5:** Pearson’s correlation coefficients between teat measurements, residual milk, milk ejection time, total milking time, and somatic cell counts.

	TWT	TCL	TCD	TET	TAD	TBsD	TBrD	TL
RM	−0.01	−0.02	−0.01	−0.03	0.18	−0.17	−0.12	0.02
TE	0.05	0.02	0.01	0.17	0.08	0.08	0.06	0.35^***^
TMT	−0.03	−0.05	0.04	0.07	−0.03	0.03	0.01	0.22^**^
SCC	0.01	0.21^**^	−0.16	−0.20^*^	−0.21^*^	−0.12	−0.02	−0.07

TWT, teat wall thickness; TCL, teat canal length; TCD, teat cistern diameter; TET, teat-end thickness; TAD, teat apex diameter; TBsD, teat basal diameter; TBrD, teat barrel diameter; TL, teat length; RM, residual milk; TE, milk ejection time; TMT, total milking time; SCC, somatic cell count. ^*^p < 0.05; ^**^p < 0.01; ^***^p < 0.0001.

## 4 Discussion

A high inter- and intra-variability between she-camels regarding VLOTS was observed. Only 30% of the tested teats required a low vacuum level to overcome the teat sphincter. For these loose teats, VLOTS was within the range reported for most tested species. Indeed, Sinapsis et al. (7) recorded an average of 16.59 ± 0.7 kPa of VLOTS in Greek Boutsiko ewes. Skapetas et al. (8) found that indigenous Greek goats required an average of 23.57 kPa to open the teat sphincter. In dairy cows, Le Du et al. (19) reported that the VLOTS was 17.0 kPa and 14.5 kPa for front and rear teats, respectively, while Weiss et al. (24) reported levels ranging between 17.8 and 21.0 kPa according to the teat position. Group 2, characterized by firm teat sphincters, accounted for 15% of the tested teats and required an average pressure of 47.13 ± 2.14 kPa to overcome the sphincter barrier, while 55% of the teats did not open even when the vacuum exceeded 70 kPa. Similar observations were reported in buffaloes (25). In this experiment, a continuously increasing vacuum (without a liner or pulsation) induced milk flow in only 40% of tested teats at a vacuum level of up to 45 kPa, while the remaining 60% exhibited no milk flow. The aforementioned studies reported a wide variation in VLOTS across species, suggesting that genetic factors may partially explain this variability. Except for the study on VLOTS in buffaloes (25), the authors suggested reducing the milking vacuum for machine milking to improve teat conditions and reduce the residual milk (7). Weiss et al. (24) reported that the initial force required for the start of the milk flow was greater than the force needed to keep the teat sphincter open during milking. In our study, most teats did not open under excessive vacuum levels, even though milking settings recommended for similar conditions were set at 48 kPa (26). This suggests that the combined force of external vacuum and increased internal pressure from milk ejection facilitates teat sphincter opening in camels. Therefore, it is important to stimulate milk ejection before applying the milking liner to prevent excessive vacuum pressure on the teats. Parity and teat position had no effect on VLOTS in our study, consistent with the findings of Sinapis et al. (7) and Skapetas et al. (8), who reported that parity did not influence VLOTS in ewes and goats. Moreover, Weiss et al. (24) recorded that the vacuum needed to open the teat canal at the start and at the cessation of the milk flow did not differ significantly between teat positions. Nevertheless, Le Du et al. (19) reported that rear teats required less vacuum to open the sphincter and were easier to milk in cows (Table 5).

Longitudinal ultrasound scans of camel teats revealed an outer layer of homogeneous hyperechoic tissue at the periphery, representing the teat epidermis, followed by an echogenic inner layer corresponding to the teat wall (Figure 1). Between the two teat walls lies an anechoic region representing the teat cistern, which is divided into two compartments by the teat cistern wall. These two halves of the teat cistern are drained externally through two or sometimes three teat canals, which may appear in the same layout or, in some cases, only one is visible in the scanned section. In the first investigation, the easiest teats to milk had a thin wall and a shorter teat canal as observed in dairy cows (19). Unexpectedly, VLOTS was strongly correlated with milk yield (*r* = 0.4; *p* < 0.0001 and *r* = 0.33; *p* < 0.01, respectively, for morning and daily milk yield), and camel with hard opening teats produced significantly more milk. This contrasts with findings in small ruminants (7, 8) and dairy cows (19, 24). However, these studies reported increased stripping milk with increased VLOTS, suggesting a selection of animals that require lower VLOTS. Further studies are needed in camels to evaluate the relationship between VLOTS and milk flow parameters to better understand milking efficiency and optimize dairy management practices.

Different methods have been tested for measuring the teats’ reaction to machine milking by several researchers. Although ultrasonography with warm water bath method has proven to be an efficient, non-invasive technique detecting minimal changes in the teat tissue in several species (9, 11, 17, 27, 28), the cutimeter method has been recommended for measuring changes in teat thickness and has been tested in most dairy species including cows (11, 20, 29), ewes (7, 21, 27), and goats (8, 30, 31). To the best of our knowledge, this is the first study to tackle machine-induced changes in the teat tissue of the dromedary camels using both methods. Ultrasound teat scanning in a water bath prevented tissue deformation, as reported in previous studies (17, 24, 28). The results have shown that the milking machine caused an increase in the teat wall thickness (TWT) and in the teat canal length (TCL), against a decrease in teat cistern diameter (TCD) and teat apex diameter (TAD) observed by ultrasound. Teat-end thickness (TET), also called teat tissue density, measured by cutimeter decreased significantly after milking in our dairy camels. Using an ultrasound technique, similar results have been reported in small ruminants (30) and Holstein cows (32, 33).

**Figure 3:**
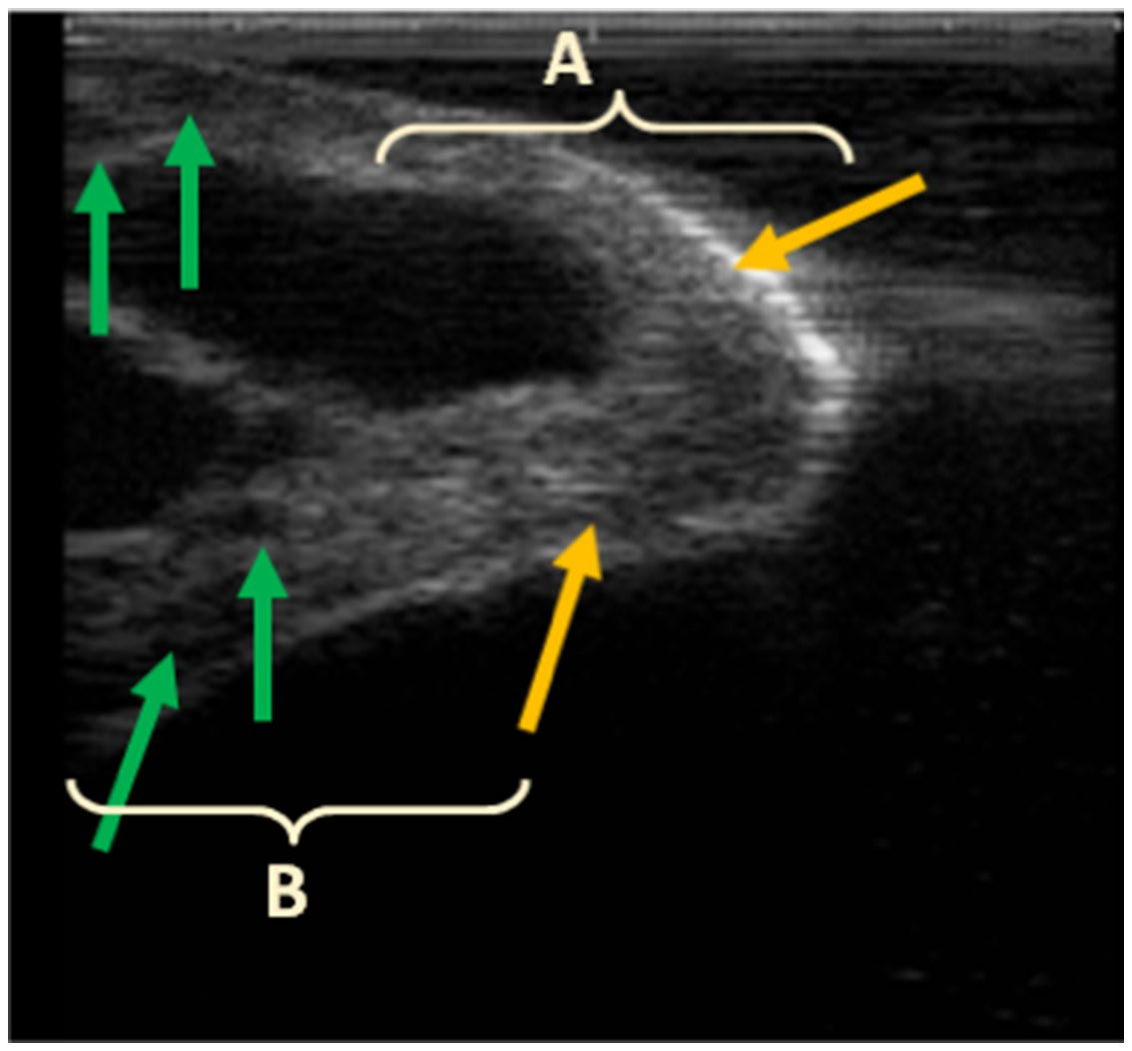
Post-milking ultrasound of the teat apex (A) and barrel (B); hyperechoic area at the tip of the teat (yellow arrows) and fluid accumulation in the teat barrel, seen as small hypoechoic areas in the teat wall (green arrows).

**Figure 4:**
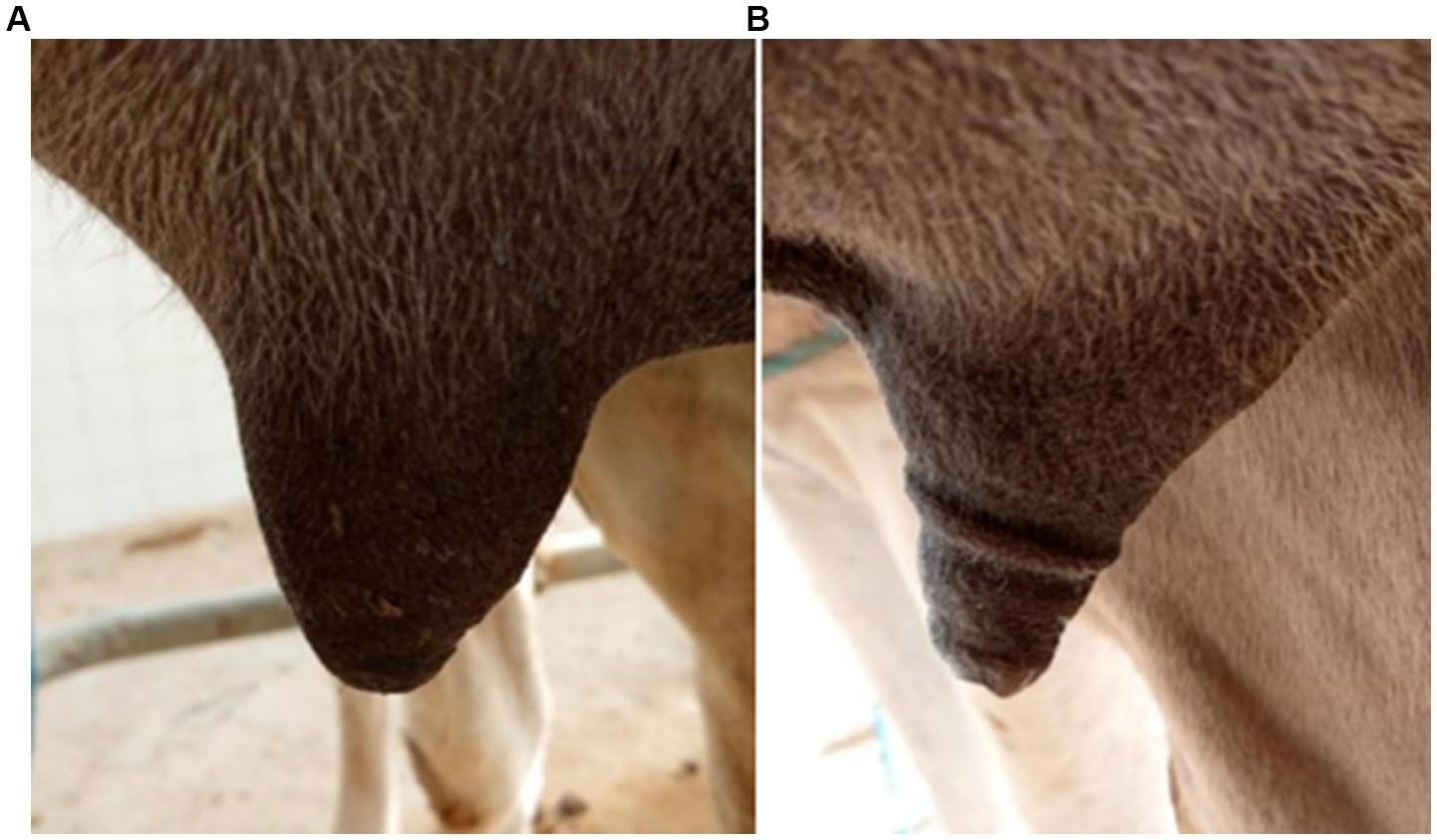
Visual appearance of camel teats before **(A)** and immediately after milking **(B)**.

Indeed, Gašparík et al. (9) reported that the milking-induced changes in teat tissue have a complex interaction with the teat measurements in dairy cows. They revealed that a thick teat barrel and thick teat apex decreased in thickness during milking, whereas a thin teat barrel and thin teat apex thickness increased. In this case, the liner encloses the teat, acting as a physical boundary that either restricts or facilitates the expansion of teat tissue. Similarly, in our study, the pre-milking dimensions of camels’ teats were within the range of thick teat barrels (>26,5 mm). The length of the teat canal increased, while the diameter of the teat apex and teat-end thickness decreased, indicating that the teat tip endured longitudinal stretching due to the vacuum suction and the weight of the milking claw attached to the teats. We hypothesize that the physical response observed in the camels’ teats under the applied milking conditions suggests that the teats were drawn into the liner under the effect of high vacuum (48 kPa) up to the point of closure, which varies from one teat to another depending on its morphology and measurements. This resulted in two main effects: (i) the formation of compression rings at the teat base, which also varied according to the teat length and diameter of the teat, and (ii) the stretching of the teat tip, which was pulled into the pulsation chamber. This led to an elongation of the teat canal and a reduction in the apex diameter, as observed via ultrasound, and consequently, a decrease in teat tip tissue density, as measured by the cutimeter. Incidentally, ultrasound scans clearly show that the accumulation of lymphatic or blood-derived fluid and the formation of edema occur above the contact line between the teat and the liner (Figure 3), which causes the formation of compression rings at the attachment point of the liner to the teat (Figure 4). Ayadi et al. (34) also reported that teat length increased after machine milking at high vacuum level (50 kPa) approximately 10.3 to 14.7% indicating that camels’ teats tend to stretch after milking. This contrasts with observations in cattle, where fluid accumulation is typically concentrated at the teat tip (11, 28).

Although the milking liner affected significantly the teat’s dimensions, no correlation was recorded between residual milk and teat measurements. Residual milk averaged at 0.57 ± 0.1 L and did not exceed 15% of total milk production. This suggests that the milking equipment and settings used in this experiment emptied efficiently and evenly the udder of camels with different teat dimensions and shapes (2). In this study, only teat length was positively correlated with time to milk ejection and total milking duration. This implies that longer teats need more stimulation time, consequently, more time to finish the milking procedure. In a previous study, teat length was associated with the massaging efficiency of the liner, which could be not sufficient on the teat (2) since the animal was only under the stimulation of the milking liner. This suggests that long teats penetrate too deeply into the pulsation chamber, reducing their exposure to the massaging effect of pulsation. As reported by Gleeson et al. (22), liner design plays a crucial role in teat tissue response and milking efficiency, with improper liner fit leading to inadequate milking and potential teat tissue damage. Similarly, Kaskous and Pfaffl (35) emphasize the importance of matching liner design to teat morphology to prevent issues such as teat congestion and compromised blood flow. These observations indicate that the liner used in this study may not be optimally suited for the teat conformation of these camels, potentially affecting milk ejection efficiency.

The teat plays a crucial role as a barrier between the animal’s internal environment, which must be protected, and the external environment, where potential contamination can occur. It is continuously exposed to various stressors, including the mechanical effects of milking. Maintaining teat integrity is essential as it directly influences milk quality and contributes to reducing the risk of elevated somatic cell counts and subsequent mastitis development. The increase in thickness may affect the teat defense mechanisms, raising the likelihood of new intramammary infections (11). In the current study, we found an average somatic cell count (SCC) of 149.6 10^3^ cells/mL, ranging between 37.5 10^3^ cells/mL and 287.5 10^3^ cells/mL. These values reflect a good mammary health status in the camels used throughout this study (36–38). However, a positive correlation between SCC, TET, and TCL was noted. Moreover, teat ring formation was positively associated with SCC (*r* = 0.33, *p* < 0.01). This implies the potential effects of machine milking on camel’s udder health, particularly those with thicker teat ends.

The findings of this study indicate that in dromedary camels, the teat sphincter response to milking vacuum requires careful consideration. As most camels require high vacuum to open the teat sphincter, the need for pre-stimulation seems more crucial in this species. The observed teat morphology, including a potentially thicker teat-end wall and a more resistant sphincter, may influence the effectiveness of machine milking. This suggests the need to reassess the applied vacuum levels to optimize milking efficiency and ensure proper teat opening. Furthermore, the impact of overmilking should be investigated as prolonged exposure to vacuum can exacerbate teat tissue stress and increase the risk of teat-end congestion. Given the strong relationship between vacuum level, teat tissue characteristics, and residual milk, further research is necessary to establish species-specific milking parameters that improve milk removal efficiency while preserving udder health in camels.

## Data Availability

The raw data supporting the conclusions of this article will be made available by the authors, without undue reservation.
